# Basic and clinical immunology – 3011. Evaluation of selected parameters of cellular immunity at diagnosis in children with osteosarcoma

**DOI:** 10.1186/1939-4551-6-S1-P187

**Published:** 2013-04-23

**Authors:** Katarzyna Markiewicz, Krzysztof Zeman, Agata Kozar, Maria Golebiowska Wawrzyniak, Wojciech Wozniak

**Affiliations:** 1 Department of Clinical Immunology, Institute of Mother and Child, Poland; 2 Department of Pediatrics and Preventive Cardiology and Immunology in Childhood, Medical University of Lodz, Poland; 3 Department of Oncological Surgery, Institute of Mother and Child, Poland

## Background

Osteosarcoma is the most frequent primary malignant bone tumour, mainly affects children in the first and second decade of life. Causes of the disease are still unknown and reaction of the immune system on its development is very individual. Specific antigen-dependent cellular immunity plays an important role in this process. Cytotoxic T lymphocytes, NK , NKT and Tγδ lymphocytes are engaged directly in destruction of the tumour, helper T lymphocytes and indirectly B lymphocytes are of special importance.

The aim of study was evaluation of selected elements of cellular immunity in children with osteosarcoma at diagnosis level.

## Methods

Study was performed on the group of 44 children with osteosarcoma, aged from 6 to 20 years (median 15.0 years). The control group formed 22 children in the same age (median 14,5 years) without a diagnosis of neoplastic disease and active inflammatory state. T lymphocytes with their subpopulations, lymphocytes B, NKT and NK cells from peripheral blood were analyzed by flow cytometry method. Examinations were performed before the therapy - in diagnostic period.

## Results

Lower number of lymphocytes population in children with osteosarcoma compared to the control group was observed. The differences concerned lymphocytes CD3+(1609,0 vs 3038,0 kom/μl, p<0,001) CD4+(598,0 vs 1071,0 kom/l; p<0,001) and CD8+ (386.0 vs. 866.0 cells/μL; p<0.001), activated T lymphocytes CD3+/HLA-DR+(39.0 vs. 81.0 cells/μL; p<0.025), B lymphocytes CD19+(205.0 vs. 381.0 cells/μL; p<0.025) and NK cells (161.0 vs. 339.0 cells/μL; p<0.005).

## Conclusions

12) General analysis of peripheral blood without differentiation of lymphocytes subpopulations is insufficient to determine disturbances which are forming in the immune system of patients with the developing neoplastic disease. 2.) The number and percentage of lymphocytes T with their subpopulations, lymphocytes B and NK cells is decreased in patients with osteosarcoma at diagnosis.

